# Supplementary material for: Influence of Concentration, Surface Charge, and Natural Water Components on the Transport and Adsorption of Polystyrene Nanoplastics in Sand Columns
Source: Nanomaterials (Basel). 2024 Mar 15;14(6):529. doi: 10.3390/nano14060529 (PMC10974996; doi:10.3390/nano14060529)
Supplement: Supplementary file 1 [file nanomaterials-14-00529-s001.zip › nanomaterials-2892896-supplementary.pdf]

Supporting information (SI)

for the article entitled:

Influence of Concentration, Surface Charge, and Natural Water Components on the Transport and Adsorption of Polystyrene Nanoplastics in Sand Columns

Gabriela Hul [1,*], Hande Okutan [2,3], Philippe Le Coustumer [2,4], Stéphan Ramseier Gentile [5], Stéphane Zimmermann [5], Pascal Ramaciotti [5], Pauline Perdaems [5] and Serge Stoll [1]

1 Department F.-A. Forel for Environmental and Aquatic Sciences, Institute for Environmental Sciences, University of Geneva, 1205 Geneva, Switzerland
2 Ecole Doctorale, Université de Bordeaux Montaigne, 33607 Pessac, France
3 Department of Geological Engineering, University of Mugla Sitki Kocman, Mugla 48260, Türkiye
4 Bordeaux Imaging Center CBRS—INRAE—INSERM, Université de Bordeaux, 33000 Bordeaux, France
5 SIG—Industrial Services of Geneva, 1211 Geneva, Switzerland
* Correspondence: gabriela.hul@unige.ch; Tel.: +41-22-379-03-31

Nanoplastics characteristics

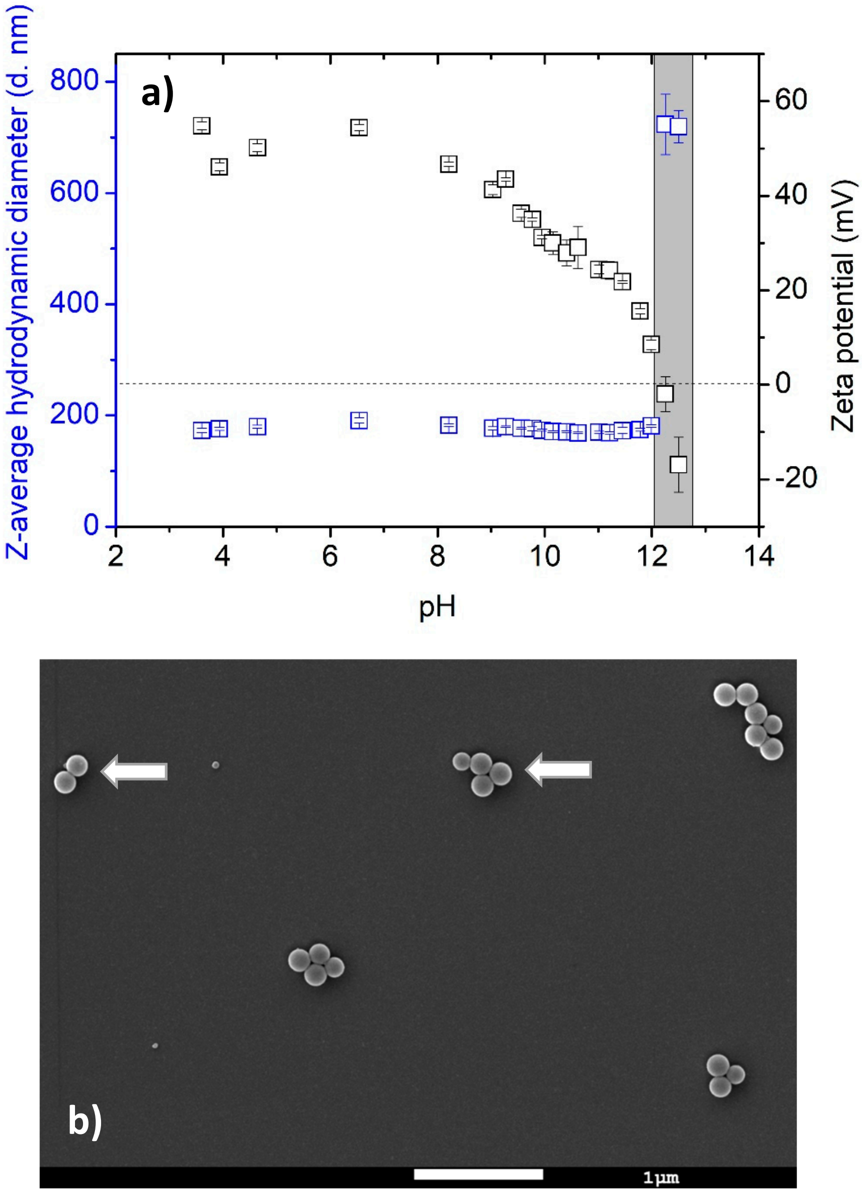

Figure S1. a) ζ potential and z-average hydrodynamic diameter variations of amidine polystyrene (PS) latex nanoplastics (NPLs) (50 mg L^{-1}) as a function of pH in ultrapure water. The point of zero charge (PZC) is found at pH of 12.2 ± 0.1 and the grey zone corresponds to destabilization zone. b) SEM image of amidine PS latex NPLs dispersed in ultrapure water; pH < pH_{PZC}, [PS NPLs] = 20 mg L^{-1}. White arrows some NPLs which have been measured manually to confirm their size ranges. This micrography exhibits NPLs forming small linear aggregates.

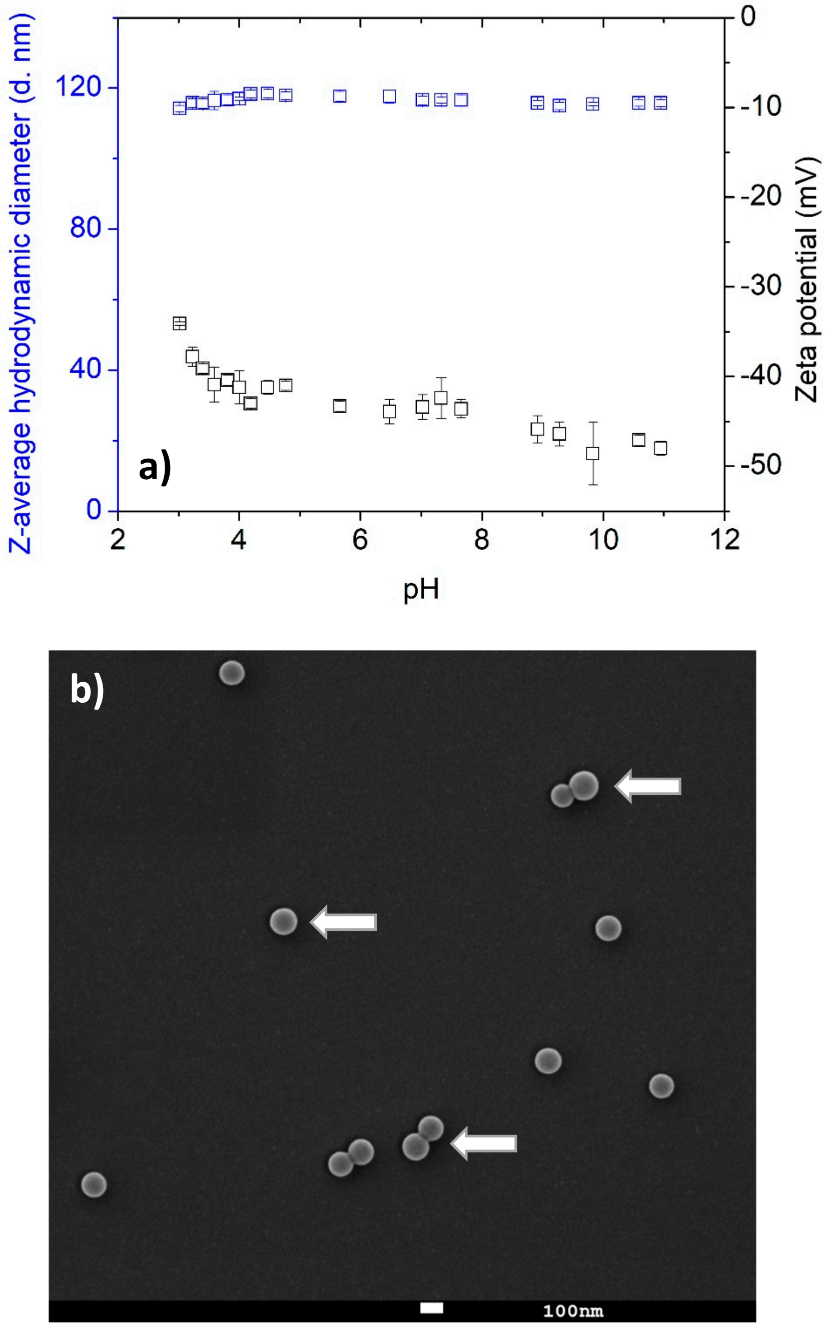

Figure S2. a) ζ potential and z-average hydrodynamic diameter variations of sulfate PS latex NPLs (50 mg L^{-1}) as a function of pH in ultrapure water. b) SEM image of sulfate PS latex NPLs dispersed in ultrapure water; pH < pH_{PZC}, [PS NPLs] = 20 mg L^{-1}. NPLs are well dispersed (white arrows) which allows precise size measurements.

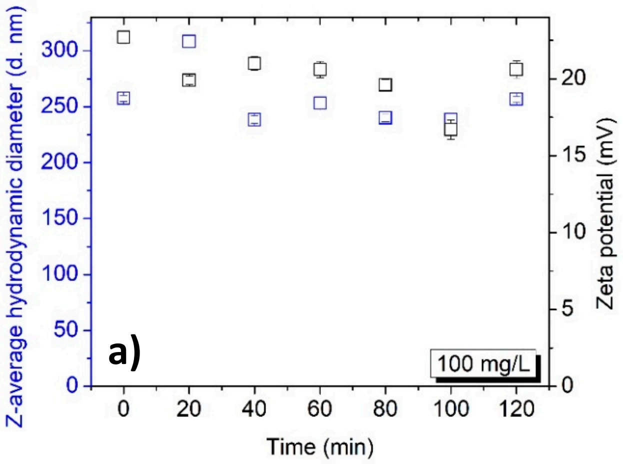

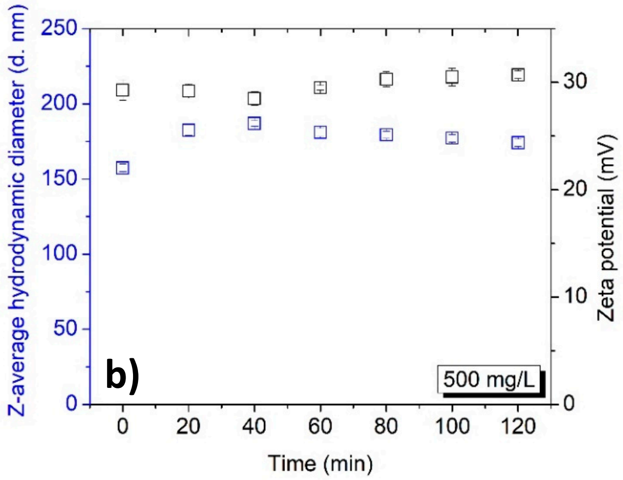

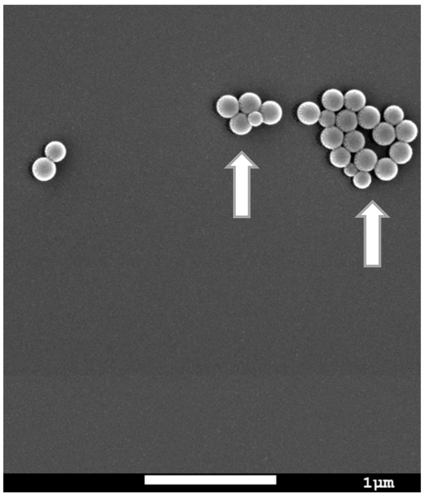

Figure S3. ζ potential and z-average hydrodynamic diameter variations of 100 mg L^{-1} (a) and 500 mg L^{-1} (b) amidine PS latex NPLs as a function of time in Geneva Lake water. c) SEM image of amidine PS latex NPLs dispersed in Geneva Lake water; pH of 8.6 ± 0.2, [NPLs] = 50 mg L^{-1}. NPLs aggregation (white arrows) occurred due to use of diluted NPLs concentration.

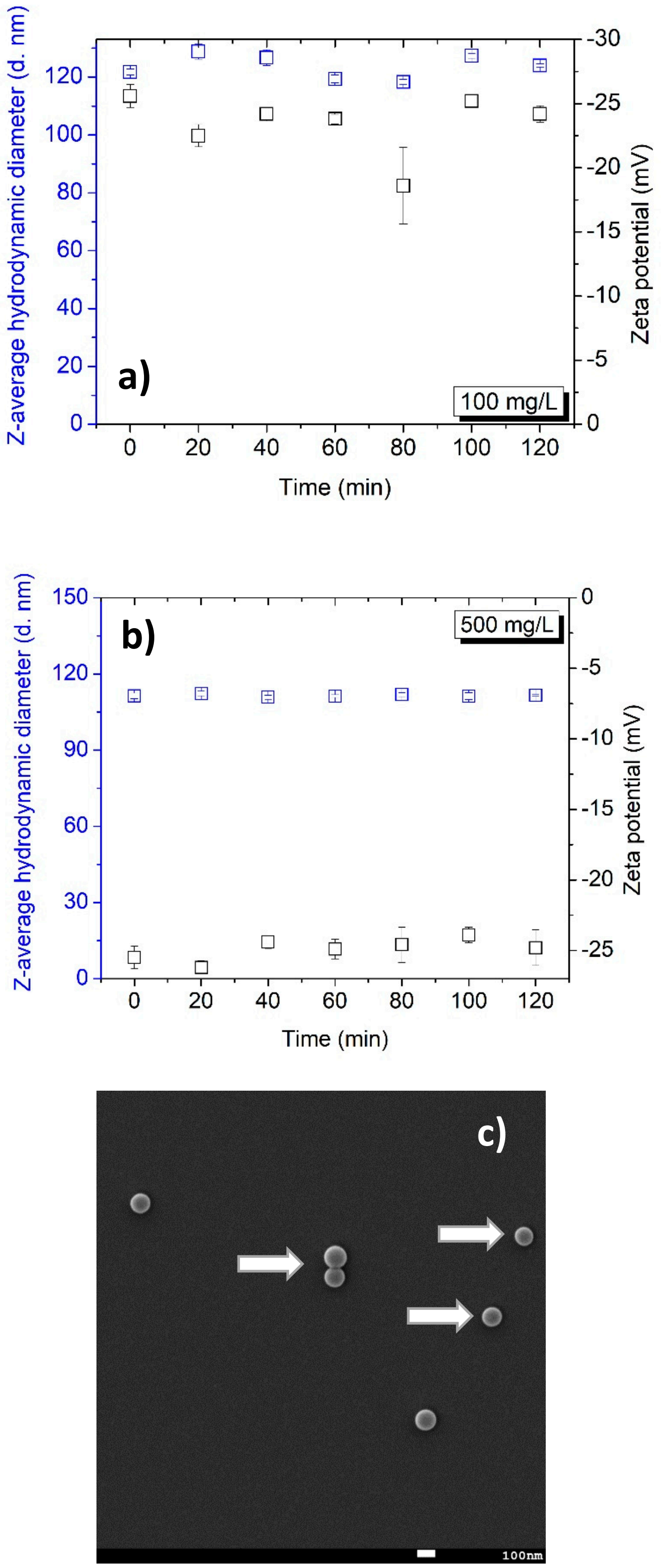

Figure S4. ζ potential and z-average hydrodynamic diameter variations of 100 mg L^{-1} (a) and 500 mg L^{-1} (b) sulfate PS latex NPLs as a function of time in Geneva Lake water. c) SEM image of sulfate PS latex NPLs dispersed in Geneva Lake water (white arrows); pH of 8.6 ± 0.2, [NPLs] = 20 mg L^{-1}.

Sand grains characteristics provided by the Industrial Services of Geneva (SIG)

Grain size (mm)	Volume proportion of particles in the range (%)	Cumulative distribution (%)	Sphericity	Aspect ratio	Convexity
0 - 0.75	0.05	0.05	0.785	0.660	0.979
0.75 – 1	0.00	0.05	0.806	0.581	0.992
1 – 1.2	0.01	0.06	0.567	0.328	0.979
1.2 – 1.5	0.07	0.13	0.577	0.310	0.982
1.5 – 2	1.54	1.67	0.708	0.437	0.986
2 – 2.38	7.26	8.93	0.774	0.529	0.988
2.38 – 2.83	24.74	33.67	0.823	0.622	0.989
2.83 – 3.36	40.64	74.31	0.845	0.692	0.989
3.36 – 4	22.98	97.29	0.848	0.728	0.987
4 – 4.76	2.67	99.96	0.826	0.761	0.982
> 4.76	0.04	100	0.654	0.742	0.956

Table S1. Sand grains characteristics determined with Camsizer (Retsch). The majority of sand grains have sizes ranging from 2.4 to 4 mm, an average roughness equal to 0.83 and an average convexity almost equal to 1. Roughness and convexity calculations suggest that surface of sand grains can have small cavities and do not have any protrusions.

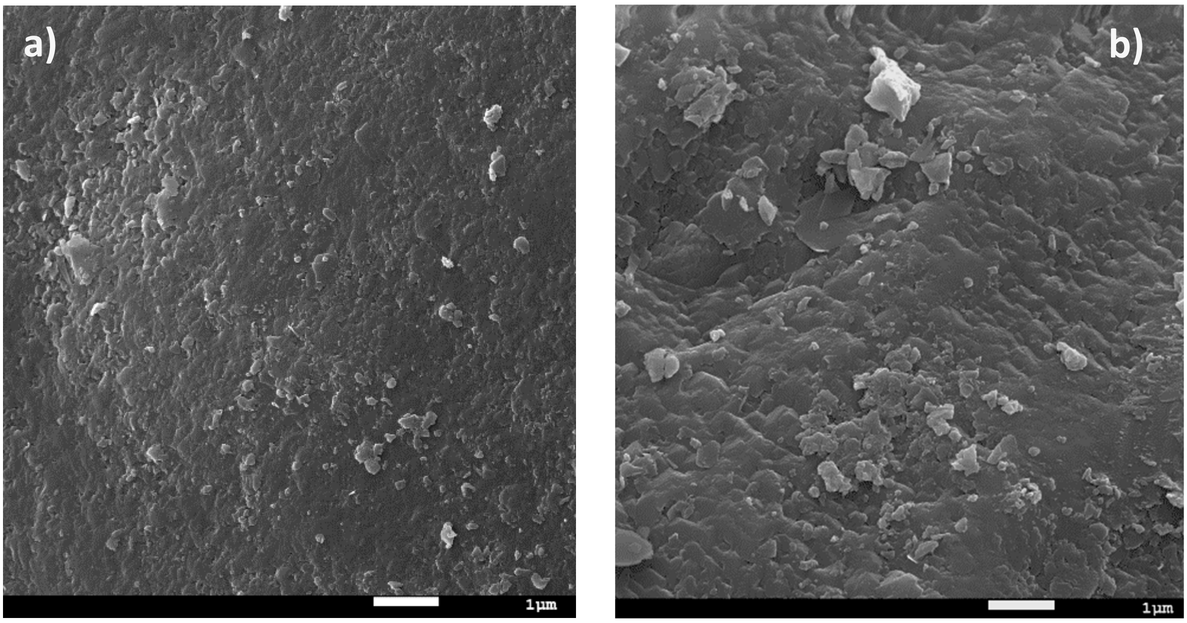

Figure S5. SEM images of pristine sand surface after 2 h of pre-conditioning with ultrapure water (a) and Geneva Lake water (b). The surface of the sand is very heterogenous and development of biofilm in the case of Geneva Lake water was not observed.

Geneva Lake water characteristics

Name of element	Unit	Value
**Total organic carbon**	mg L^{-1}	1.06 ± 0.17
**Sodium (Na^{+})**	mg L^{-1}	7.10 ± 0.21
**Potassium (K^{+})**	mg L^{-1}	1.48 ± 0.11
**Calcium (Ca^{2+})**	mg L^{-1}	44.16 ± 1.17
**Magnesium (Mg^{2+})**	mg L^{-1}	4.44 ± 0.64
**Fluorine (F^{-})**	mg L^{-1}	0.11 ± 0.05
**Chloride (Cl^{-})**	mg L^{-1}	10.68 ± 0.21
**Sulfate (SO_4^{2-})**	mg L^{-1}	39.57 ± 1.63

**Physicochemical parameters**		
**pH**	-	7.23
**Conductivity**	µS cm^{-1} 20°C	262

Table S2. Concentrations of major elements in Geneva Lake water and physicochemical characteristics of Geneva Lake water samples.

Fluorescent tracer experiments

Fluoresceine sodium salt (Sigma Aldrich, Switzerland) was used as an inert dye tracer. 1 g L^{-1} stock solution was prepared by dissolving a given amount of powder in ultrapure water and it was further used to prepare solutions of lower concentrations. The stock solution and its dilutions were not submitted to any kind of purification and immediately used. Tracer column experiments were performed as described above. A DR3900 spectrophotometer (Hach Lange, Switzerland) was used to determine dye concentrations in the effluent. First, a relation between dye concentration and absorbance was established by measuring a series of samples of known concentration (0 - 20 mg L^{-1}) at the wavelength of 460 nm. A calibration curve was then established according to the Beer-Lambert law, and it was subsequently used to determine dye concentrations in the effluent. All measurements were performed in 1 cm path length quartz cuvette at a room temperature. The calibration curve and experimental BTC are presented in Figure S6 and S7. An analytical solution of 1D Advection-Dispersion equation for instantaneous injection in a semi-infinite medium proposed by Kreft and Zuber (1978) was used to derive values of porosity, dispersion coefficient and mean velocity (Kreft and Zuber 1978). The porosity and hydrodynamic characteristics were determined by fitting experimental and model BTCs using Excel's solver extension.

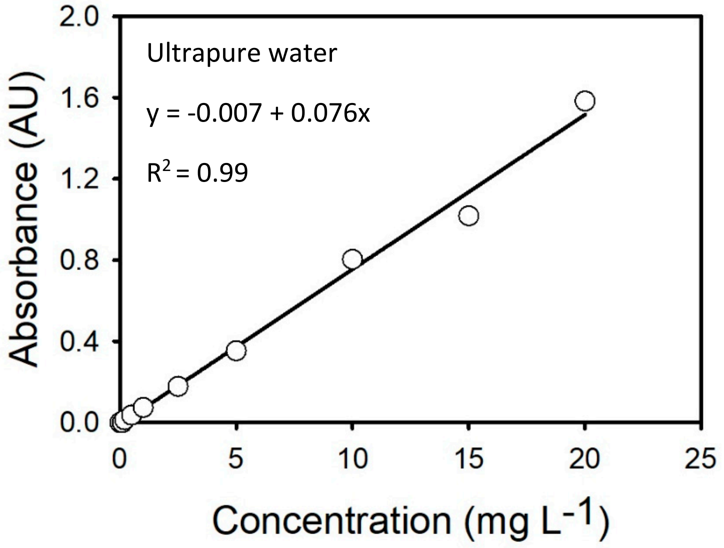

Figure S6. Calibration curve established for fluorescent tracer. Intercept, slope, and correlation coefficient are equal to -0.007, 0.076 and 0.99, respectively.

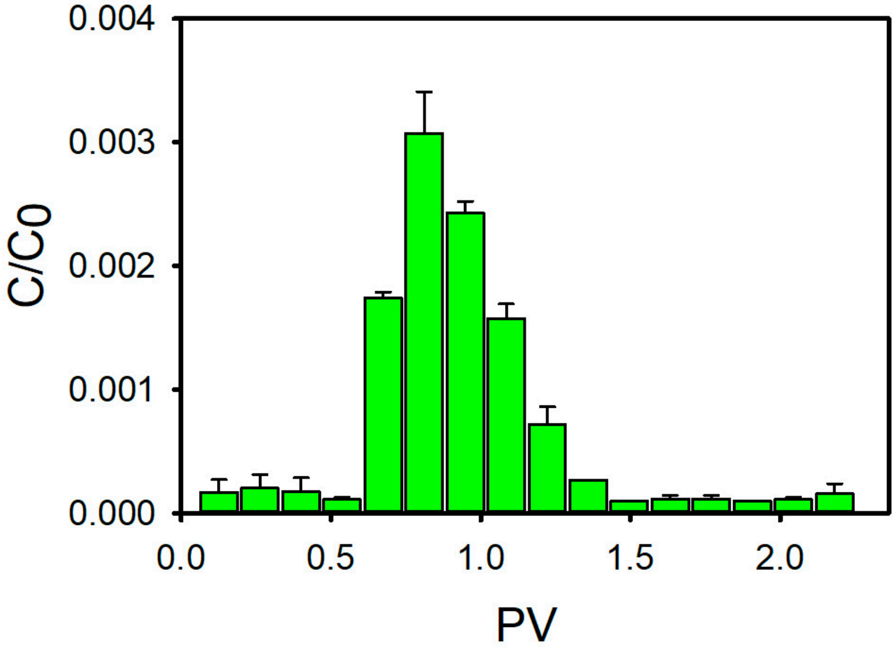

Figure S7. Breakthrough curve of fluorescent tracer.

Calibration curves established for different nanoplastics dispersions

NPLs concentrations in the effluent were determined using a Hach Turbidimeter model TU5200 (Hach Lange, Switzerland). To evaluate how water turbidity changes with NPLs concentration, instrumental responses for several samples having NPLs concentrations comprised between 0.1 and 50 mg L^{-1} were measured, and calibration curves were established. Samples were stabilized for 15 min before turbidity check. All measurements were performed at room temperature. Simultaneously, NPLs stability in terms of z-average hydrodynamic diameter and ζ potential was verified using ZetaSizer Nano ZS. A calibration curve was established for each type of NPLs and for each type of water. The turbidity generated by the presence of sand grains and the turbidity of Geneva Lake water were not subtracted from final readings to enable comparison with real-scale experiments.

In ultrapure water, the turbidity changes linearly with concentration for both types of NPLs. For amidine PS latex NPLs, intercept, slope, and correlation coefficient of calibration curve are equal to 0.09, 2.64 and 1.0, respectively (Figure S8a). As for the sulfate PS latex NPLs, intercept, slope, and correlation coefficient of calibration curve are equal to 0.16, 0.88 and 0.99, respectively (Figure S9a). In general, both types of NPLs dispersions were stable during turbidity measurements and small aggregation and decrease in ζ potential occurred for NPLs concentrations smaller or equal to 1 mg L^{-1} (Figure S8b, S8c, S9b and S9c).

In Geneva Lake water, the turbidity also changes linearly with concentration for both types of NPLs. For amidine PS latex NPLs, intercept, slope, and correlation coefficient of calibration curve are equal to 0.54, 2.66 and 0.99, respectively (Figure S10). Regarding sulfate PS latex NPLs dispersed in Geneva Lake water, intercept, slope, and correlation coefficient of calibration curve are equal to 0.22, 1.09 and 0.99, respectively (Figure S11). During turbidity measurements, NPLs dispersed in Geneva Lake water (0.1 – 50 mg L^{-1}) were not stable and they were forming aggregates. Z-average hydrodynamic diameter of amidine PS latex NPLs was varying from 380 d. nm for the smallest concentration (0.1 mg L^{-1}) to approximately 4 μm for larger concentrations (Figure S10b). In parallel, ζ potential of amidine PS latex was negative for small NPLs concentration and turned positive for larger concentrations (> 30 mg L^{-1}) (Figure S10c). Sulfate PS latex NPLs were found more stable in Geneva Lake water and aggregates, ranging from 200 to 350 nm, occurred only in the case of smaller concentrations (<10 mg L^{-1}) (Figure S11b). Simultaneously, ζ potential oscillates around -10 ± 5 mV for

smaller concentrations (<10 mg L^{-1}) and -20 mV for higher concentrations (>10mg L^{-1}) (Figure S11c).

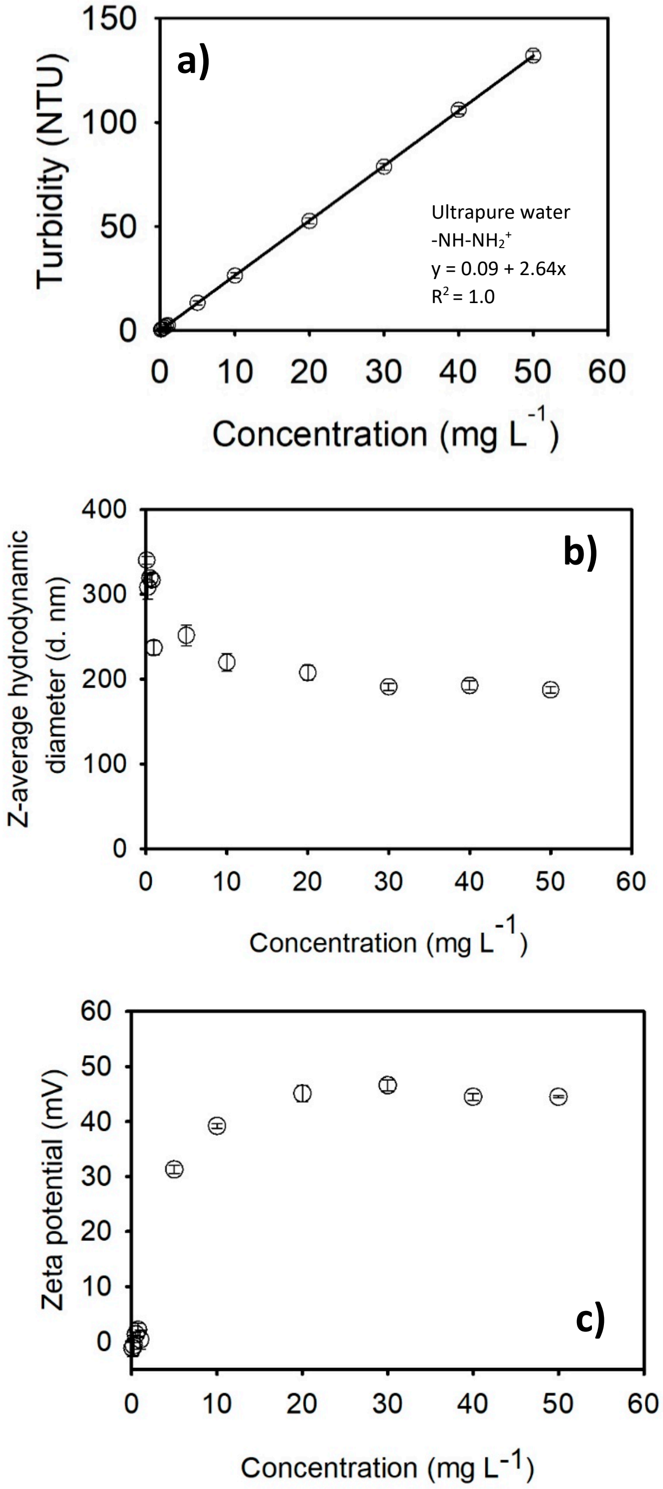

Figure S8. a) Calibration curve established for amidine PS latex NPLs dispersed in ultrapure water. Intercept, slope, and correlation coefficient are equal to 0.09, 2.64 and 1.0, respectively. Z-average hydrodynamic diameter (b) and ζ potential (c) variations of amidine PS latex NPLs as a function of NPLs concentration.

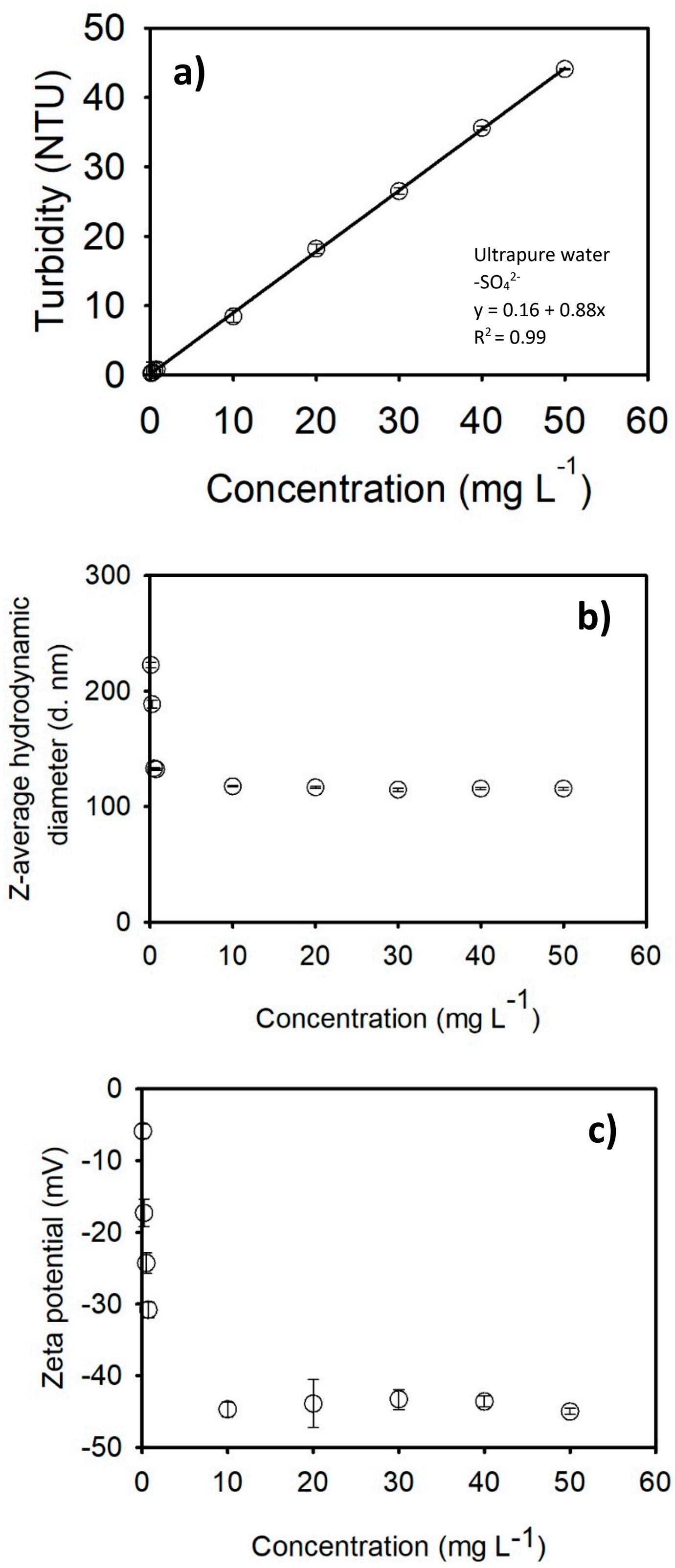

Figure S9. a) Calibration curve established for sulfate PS latex NPLs dispersed in ultrapure water. Intercept, slope, and correlation coefficient are equal to 0.16, 0.88 and 0.99, respectively. Z-average hydrodynamic diameter (b) and ζ potential (c) variations of sulfate PS latex NPLs as a function of NPLs concentration.

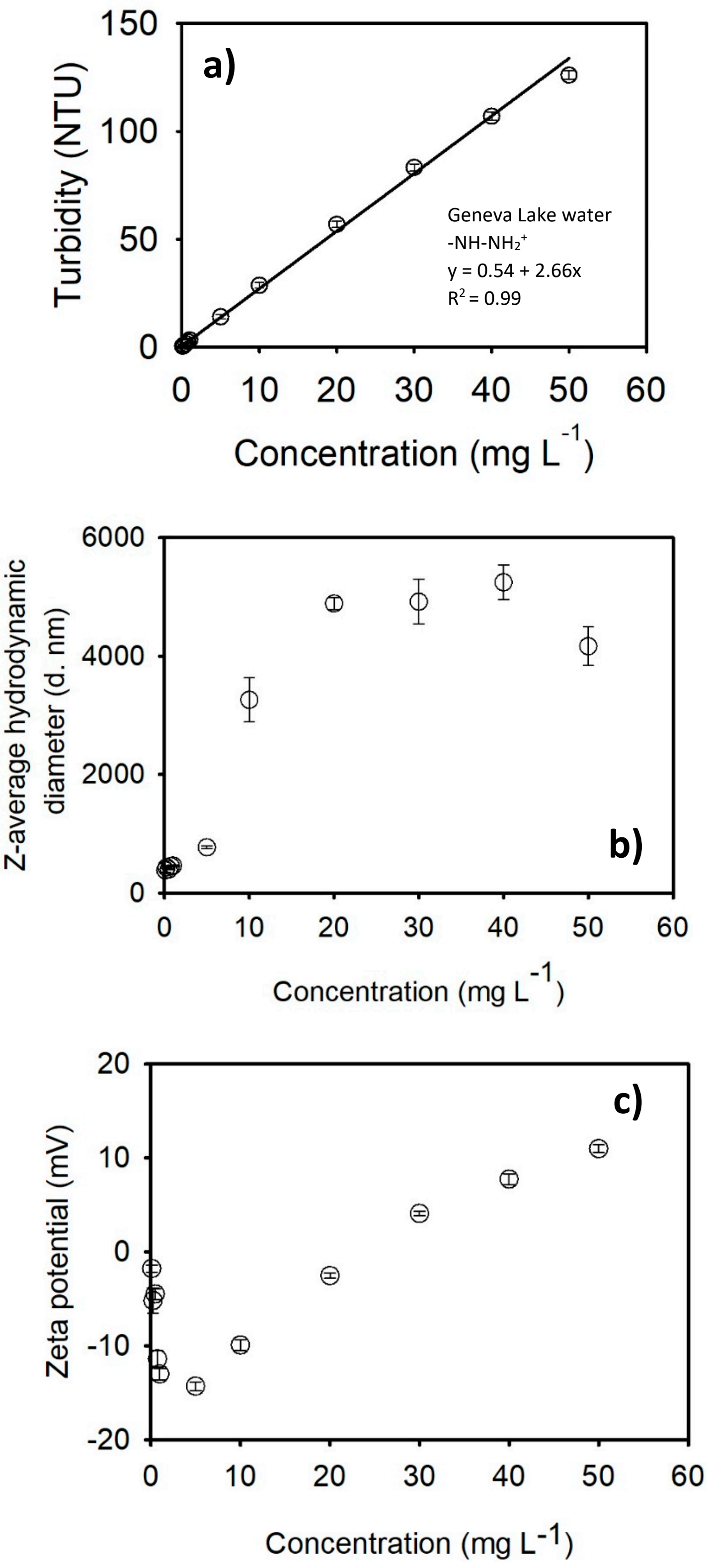

Figure S10. a) Calibration curve established for amidine PS latex NPLs dispersed in Geneva Lake water. Intercept, slope, and correlation coefficient are equal to 0.54, 2.66 and 0.99, respectively. Z-average hydrodynamic diameter (b) and ζ potential (c) variations of sulfate PS latex NPLs as a function of NPLs concentration.

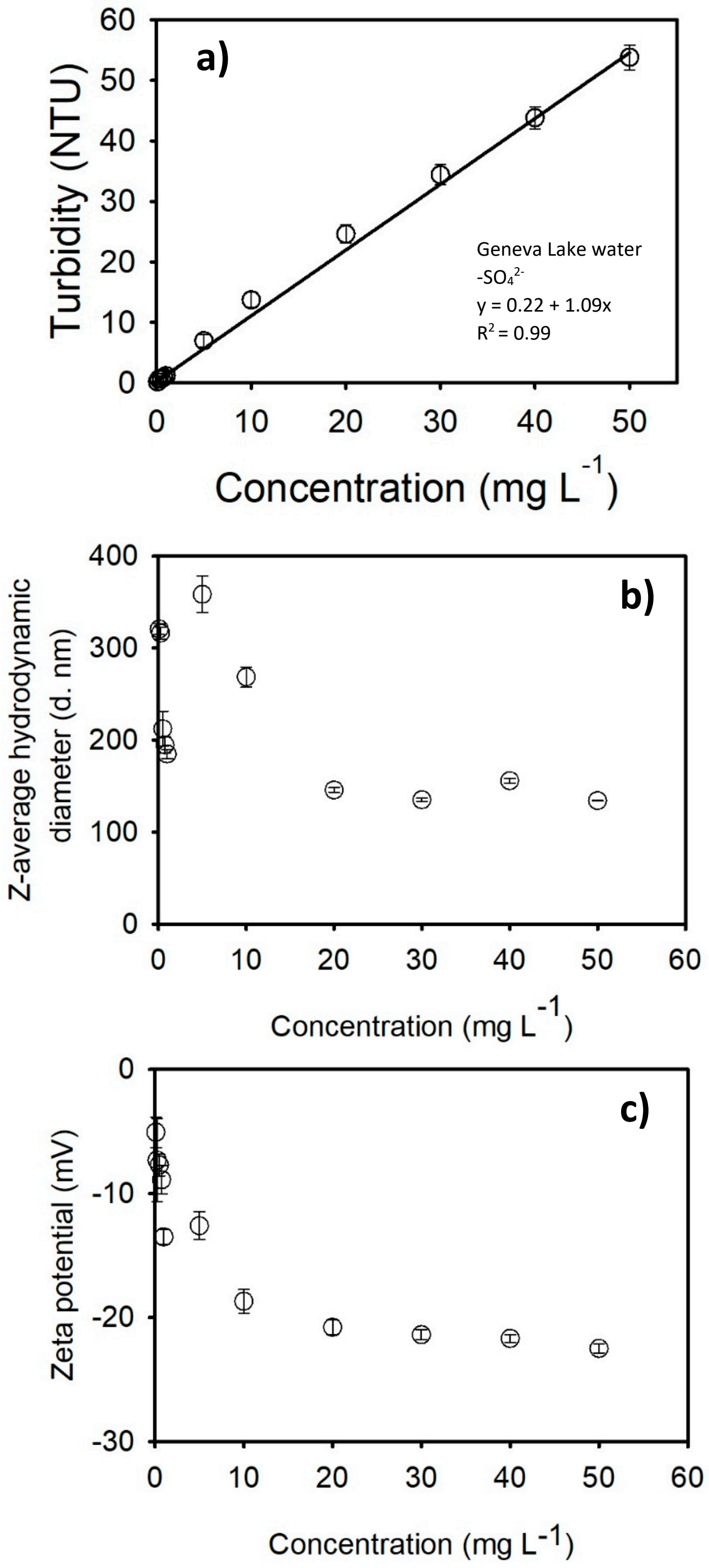

Figure S11. a) Calibration curve established for sulfate PS latex NPLs dispersed in Geneva Lake water. Intercept, slope, and correlation coefficient are equal to 0.22, 1.09 and 0.99, respectively. Z-average hydrodynamic diameter (b) and ζ potential (c) variations of sulfate PS latex NPLs as a function of NPLs concentration.

Variations in z-average hydrodynamic diameter and ζ potential of amidine polystyrene latex nanoplastics and sulfate polystyrene latex nanoplastics present in the effluent (ultrapure water)

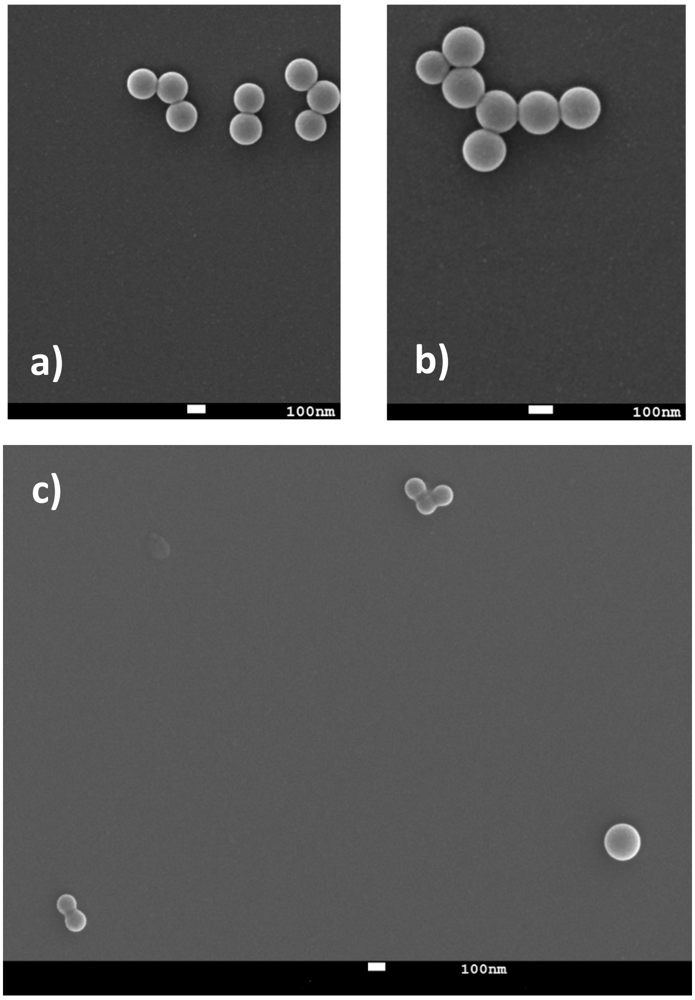

Figure S12. SEM micrography of amidine PS latex (a, b) and sulfate PS latex (c) nanoplastics present in the effluent coming from the quartz sand columns saturated with ultrapure water. Experimental conditions for SEM imaging: [NPLs] = 500 mg L^{-1}.

Variations in z-average hydrodynamic diameter and ζ potential of amidine polystyrene latex nanoplastics and sulfate polystyrene latex nanoplastics present in the effluent (Geneva Lake water)

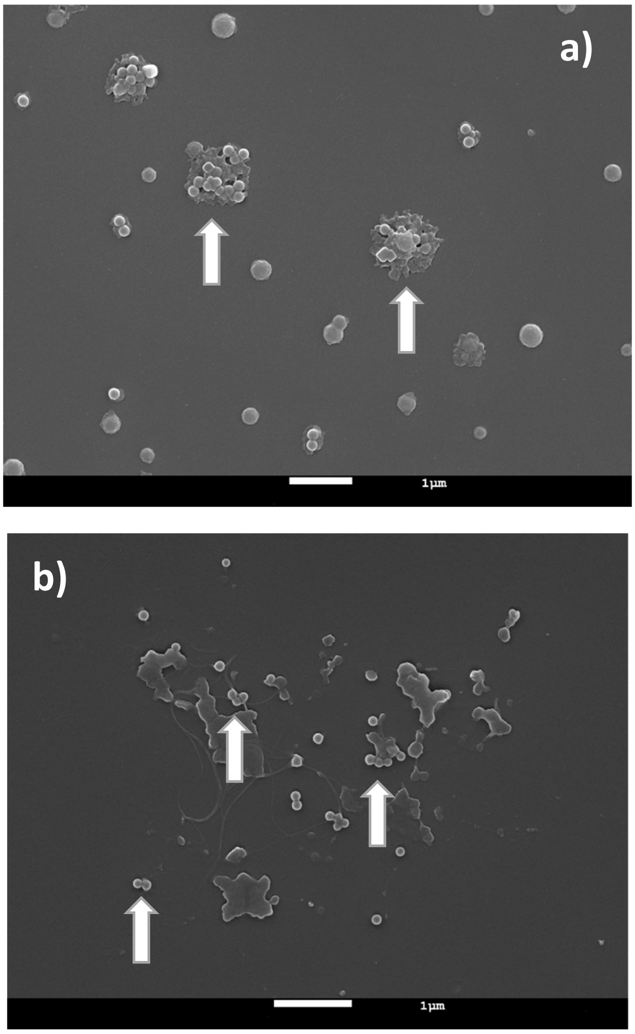

Figure S13. Amidine PS latex (a) and sulfate PS latex (b) NPLs present in the effluent coming from the quartz sand columns saturated with Geneva Lake water. Experimental conditions for SEM imaging: [NPLs] = 500 mg L^{-1}.

pH-titration curves preparation

pH-titration curves were established for both types of NPLs by titrating a 50 mg L^{-1} NPLs solution with diluted 1 M HCl or 1 M NaOH solution (Titrisol, Merck, Switzerland) in a pH range of 3.0 to 11.0. pH variations were measured using Hach Lange HQ40d portable meter (Hach Lange, Switzerland). NPLs behavior, in terms of z-average hydrodynamic diameter and zeta (ζ) potential, in changing pH conditions was characterized using ZetaSizer Nano ZS (Malvern Instruments, Worcestershire, United Kingdom). All measurements were taken approximately 15 min after pH stabilization. All samples were measured after 60s of equilibration within the machine and at an ambient temperature of 25°C. Each result corresponds to an average of 5 measurements, and each measurement was composed of 10 runs. The size of NPLs was derived using Stokes-Einstein relationship whereas the electrophoretic mobility of the NPLs was converted into a ζ potential using Smoluchowski approximation (Einstein 1905, Smoluchowski 1921).

Recovered mass and adsorption capacity calculations

BTCs were used to calculate recovered mass of fluorescent tracer and NPLs according to the following formula (Goldscheider and Drew 2007):

$$m_r = Q \int_0^{\infty} c(t) \partial t \qquad \text{(S1)}$$

where m_r is recovery mass (g), Q is flowrate (L min^{-1}), c is concentration (g L^{-1}) and t is time (min). The recovered mass expressed in percentage was subsequently used to calculate a mass of NPLs retained inside filtration column.

Adsorption capacity of sand grains was calculated according to the following equation (Tan and Hameed 2017):

$$q = \frac{QC_0 \int_{t=0}^{t=\infty} 1 - \frac{C}{C_0} dt}{m} \qquad \text{(S2)}$$

where q is adsorbed amount per unit mass of adsorbent (mg g^{-1}), Q is feed flowrate (ml min^{-1}), C_0 is feed concentration (mg ml^{-1}), m is adsorbent mass (g), and t is time (min).

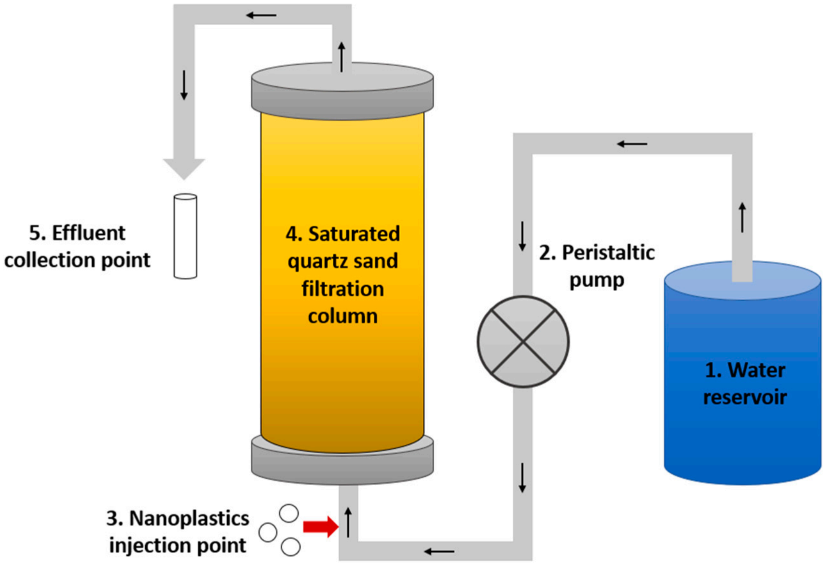

Figure S14. Experimental setup for the fluorescent tracer tests and nanoplastics transport.
